# Evaluating the quantity, quality and size distribution of cell-free DNA by multiplex droplet digital PCR

**DOI:** 10.1038/s41598-020-69432-x

**Published:** 2020-07-28

**Authors:** Miguel Alcaide, Matthew Cheung, Jack Hillman, S. Rod Rassekh, Rebecca J. Deyell, Gerald Batist, Aly Karsan, Alexander W. Wyatt, Nathalie Johnson, David W. Scott, Ryan D. Morin

**Affiliations:** 1 Department of Molecular Biology and Biochemistry, Simon Fraser University, 8888 University Drive, South Sciences Building (SSB) 7157, Burnaby, BC V5A 1S6 Canada; 2 Division of Pediatric Oncology, Hematology and Bone Marrow Transplantation, British Columbia Children’s Hospital, University of British Columbia, Vancouver, BC Canada; 3 Quebec Clinical Research Organization in Cancer (Q-CROC), Exactis Innovation and the Segal Cancer Centre, Montreal, QC Canada; 4 Cancer Genetics Laboratory, Pathology and Laboratory Medicine, British Columbia Cancer Agency, University of British Columbia, Vancouver, BC Canada; 5 Vancouver Prostate Centre and Department of Urologic Sciences, University of British Columbia, Vancouver, BC Canada; 6 Department of Medicine, Jewish General Hospital, Montreal, QC Canada; 7 Department of Lymphoid Cancer Research, BC Cancer Research Centre, Vancouver, BC Canada

**Keywords:** Cancer screening, Cancer, Biomarkers

## Abstract

Cell-free DNA (cfDNA) has become a comprehensive biomarker in the fields of non-invasive cancer detection and monitoring, organ transplantation, prenatal genetic testing and pathogen detection. While cfDNA samples can be obtained using a broad variety of approaches, there is an urgent need to standardize analytical tools aimed at assessing its basic properties. Typical methods to determine the yield and fragment size distribution of cfDNA samples are usually either blind to genomic DNA contamination or the presence of enzymatic inhibitors, which can confound and undermine downstream analyses. Here, we present a novel droplet digital PCR assay to identify suboptimal samples and aberrant cfDNA size distributions, the latter typically associated with high levels of circulating tumour DNA (ctDNA). Our assay was designed to promiscuously cross-amplify members of the human olfactory receptor (OR) gene family and includes a customizable diploid locus for the determination of absolute cfDNA concentrations. We demonstrate here the utility of our assay to estimate the yield and quality of cfDNA extracts and deduce fragment size distributions that correlate well with those inferred by capillary electrophoresis and high throughput sequencing. The assay described herein is a powerful tool to establish quality controls and stratify cfDNA samples based on presumed ctDNA levels, then facilitating the implementation of robust, cost-effective and standardized analytical workflows into clinical practice.

## Introduction

Since their initial description in 1948^1^, small DNA fragments travelling in the non-cellular component of internal bodily fluids and excretions have revolutionized numerous fields in public health and preventive medicine^2–6^. Although its origins have been a topic of controversy, cell-free DNA (cfDNA) is generally thought to arise from cellular breakdown mechanisms but also through active release from living cells^7^. Generally, cfDNA circulates in fragments ranging between 120–220 bp, or multiples thereof, with a maximum peak at 167 bp. This pattern agrees with the length of DNA wrapped around a single nucleosome, plus a short stretch of ~ 20 bp (linker DNA) bound to a histone H1^3,8^. As nucleosome positioning varies between different tissues, and in malignant neoplasms, the local pattern of fragmentation has been shown to aid in determining the predominant cell-type of origin contributing to the cfDNA pool^9,10^. The analysis of altered nucleosome fingerprints, together with outstanding advances regarding the characterization of the cfDNA methylome, hold promise regarding the detection and classification of even early-stage cancers^10,11^.

The investigation of cfDNA has several benefits that have contributed to its growing utility and popularity in medical practice^2,5,12,13^. Assays of cfDNA are nonetheless sensitive to genomic DNA (gDNA) contamination derived from lysed cells in poorly manipulated samples, cfDNA degradation and the presence of enzymatic inhibitors^3,13^. Hence, several authors have emphasized the need to standardize collection, handling, and preservation methods as well as the importance to perform consistent quality controls (QC) on isolated cfDNA^13–16^. For example, the yield of cfDNA extraction fundamentally limits the number of individual molecules that can be interrogated by any given assay and thus can significantly impact assay accuracy, precision and limit of detection. In this regard, the use of fluorometric methods is not ideal because the discrimination between cfDNA and gDNA contamination is not possible. Numerous studies have shown that cfDNA concentrations are higher in cancer patients than in healthy controls and may also provide prognostic value^3,17^, then reinforcing the need to accurately quantify cfDNA levels. Importantly, it is also becoming accepted that several environmental and physiologic factors can confoundedly contribute to the total amount of cfDNA, then undermining the utility of cfDNA concentrations in oncology^3,13^.

Factors influencing cfDNA size distribution profiles have attracted recent attention as well. Although there are contradictory reports around this topic, it is now accepted that circulating tumour DNA (ctDNA) and fetal-derived cfDNA commonly exhibit higher fragmentation than the cfDNA shed by non-neoplastic or maternal tissues, respectively^10,18–20^. In addition, recent studies have actively started to explore the diagnostic and prognostic value of cfDNA fragmentation patterns across different cancer diagnoses^10,21,22^. Here, capillary electrophoresis allows the accurate sizing and a reasonable estimation of the absolute concentration of cfDNA samples^21^. However, these methods are blind to the presence of sample impurities that can undermine downstream analyses. Enzymatic inhibitors can indeed be common in biofluids or be present in some of the chemical solutions used during cfDNA extraction^23,24^. The inhibitory effect of sample impurities can nonetheless be evaluated through quantitative real-time PCR (qPCR) spike-in experiments^14,15^. Furthermore, qPCR-based approaches have been widely used to estimate the concentration of cfDNA extracts and its integrity^14,25,26^. Such assays, however, can be negatively impacted by gDNA contamination, because it may greatly distort the ratios between short and long cfDNA-derived amplicons, and inevitably require the parallel analysis of reference samples.

In this study, we have explored the potential of droplet digital PCR (ddPCR) to establish a straightforward, robust and reproducible single-well assay for cfDNA QC that addresses some of the limitations exhibited by alternative methods. Our multiplex assay targets three fragment size ranges (73–165 bp; 166–253 bp; > 253 bp) from several olfactory receptor (OR) genes, together with the co-amplification of a customizable diploid locus (*STAT6* in or case) for the estimation of absolute cfDNA concentrations without the need of reference samples and calibration curves. We have thoroughly evaluated the performance of our ddPCR assay in a cohort comprised by 117 plasma samples collected from cancer patients and demonstrate here its utility to evaluate the quantity, quality and fragment size distribution of cfDNA samples.

## Results

### Assay resolution and estimation of total cfDNA yields

**Figure 1:**
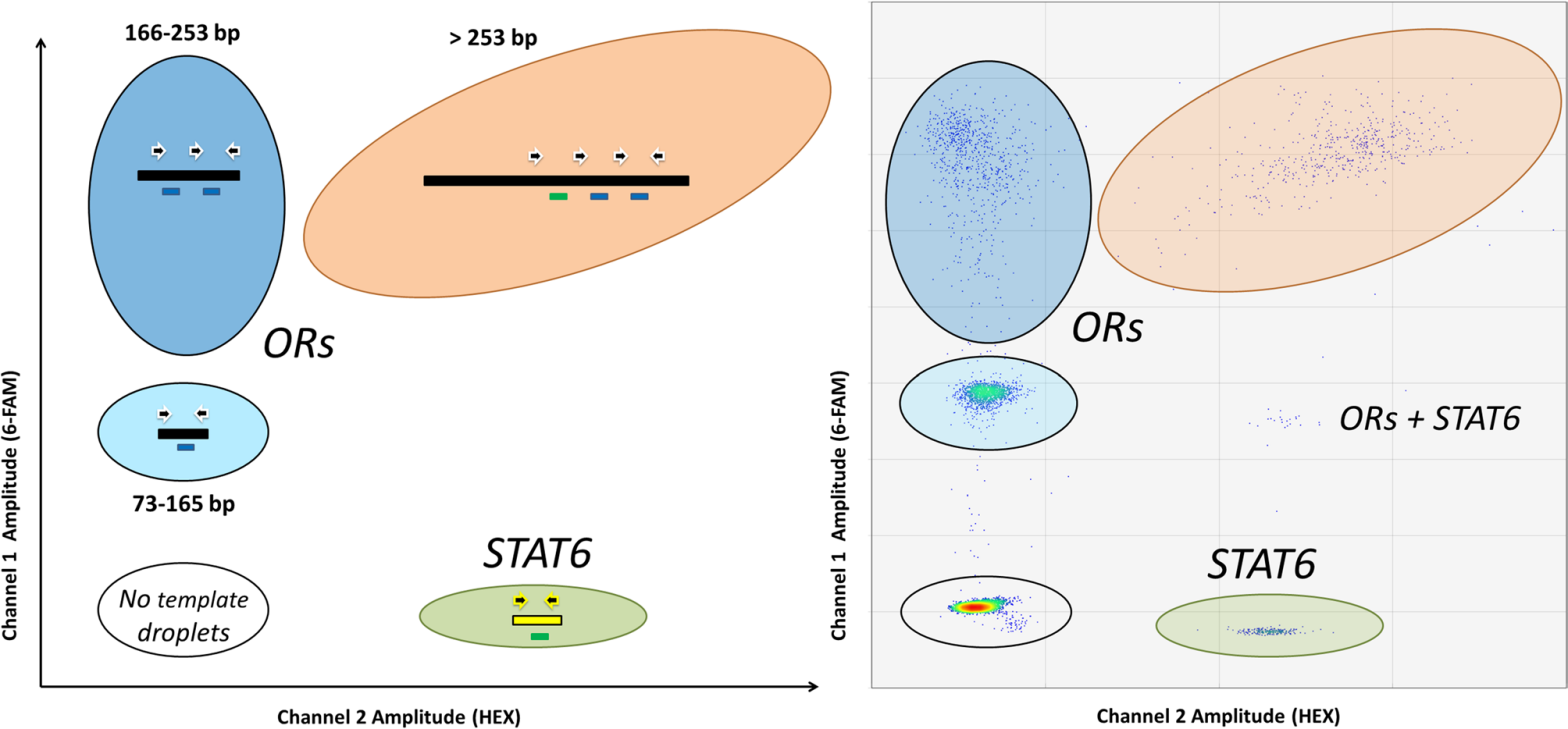
A multiplex ddPCR assay to QC cfDNA samples. Our assay permits discriminating between OR cfDNA fragments (black bars) of different sizes. Annealing sites for more than one forward primer (black arrows) and hydrolysis probe (blue bar: 6-FAM-labelled probe; green bar: HEX-labelled probe) only become available in OR cfDNA fragments exceeding 165 bp. Our assay also includes the co-amplification of a diploid locus (*STAT6*) to estimate absolute DNA concentration. A visualization of the raw data derived from the analysis of 1 ng of cfDNA can be observed in the right panel.

Our assay successfully generated five distinct fluorescence clusters, as observed in 2D fluorescence plots (Fig. 1). Three of these clusters relate to droplets carrying OR fragments of different sizes, a fourth cluster corresponds to droplets containing *STAT6* fragments and a fifth cluster was represented by droplets lacking OR or *STAT6* copies. We leveraged the number of droplets positive for the *STAT6* locus to estimate absolute cfDNA concentrations. Overall, the average cfDNA input calculated from the absolute quantification of *STAT6* copies was 1.88 ± 1.39 S.D. ng (minimum = 0.10 ng, maximum = 9.3 ng, N = 117). As stated in the methods section, we aimed at generally including between 1 and 3 ng of cfDNA per ddPCR reaction, based on previous fluorometric quantifications performed on the same set of cfDNA extractions. Our ddPCR-based estimates of cfDNA yields were in line with expectations. Small discrepancies are expected, owing to pipetting errors or variations in the volume and concentration of cfDNA extracts that were extracted and quantified by fluorometric methods months or years before. For example, a read of 20 copies/µL is equivalent to 20 copies × 22 µL (final volume of the ddPCR reaction) = 440 copies total. Assuming that 1 ng of cfDNA roughly contains 303 haploid genome equivalents, this corresponds to 440/303 = 1.45 ng of cfDNA.

The average ratio between the number of positive droplets for OR fragments versus droplets positive for *STAT6* fragments was 10.0 ± 2.6 S.D.; minimum = 5.2, maximum = 21.9, N = 117). These results may suggest the cross-amplification of additional OR loci beyond those targeted explicitly by our primers. Given the close proximity between the three targets being quantified, and the high level of sequence conservation across the regions where both primers and probes sit, we assume that every single extra OR copy being cross-amplified with our primers will permit the analysis of each one of the three fragment size distributions. Extraordinarily high ORs/*STAT6* ratios (> 15.0), observed only in six out of 117 cfDNA samples, point towards rare somatic copy number gains of these genes in some tumours. Contrarily to this finding, extensive OR copy number losses (i.e. indicated by ORs/*STAT6* ratios < 5) were not observed in our data.

### Evaluation of cfDNA fragment size distribution and assay precision

**Figure 2:**
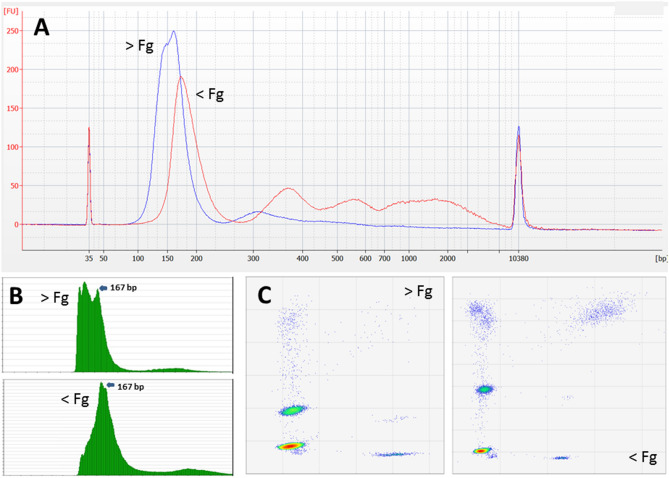
Visualizing fragment length distribution of cfDNA by electrophoretic mobility, high throughput sequencing and ddPCR. Two representative samples exhibiting low (> Fg) and “normal” (< Fg) cfDNA size distributions are compared through three comparable methods. (**A**) Analysis of the electrophoretic mobility of cfDNA fragments, (**B**) fragment length distribution of mapped sequencing reads or (**C**) using our multiplexed ddPCR assay described herein.

**Figure 3:**
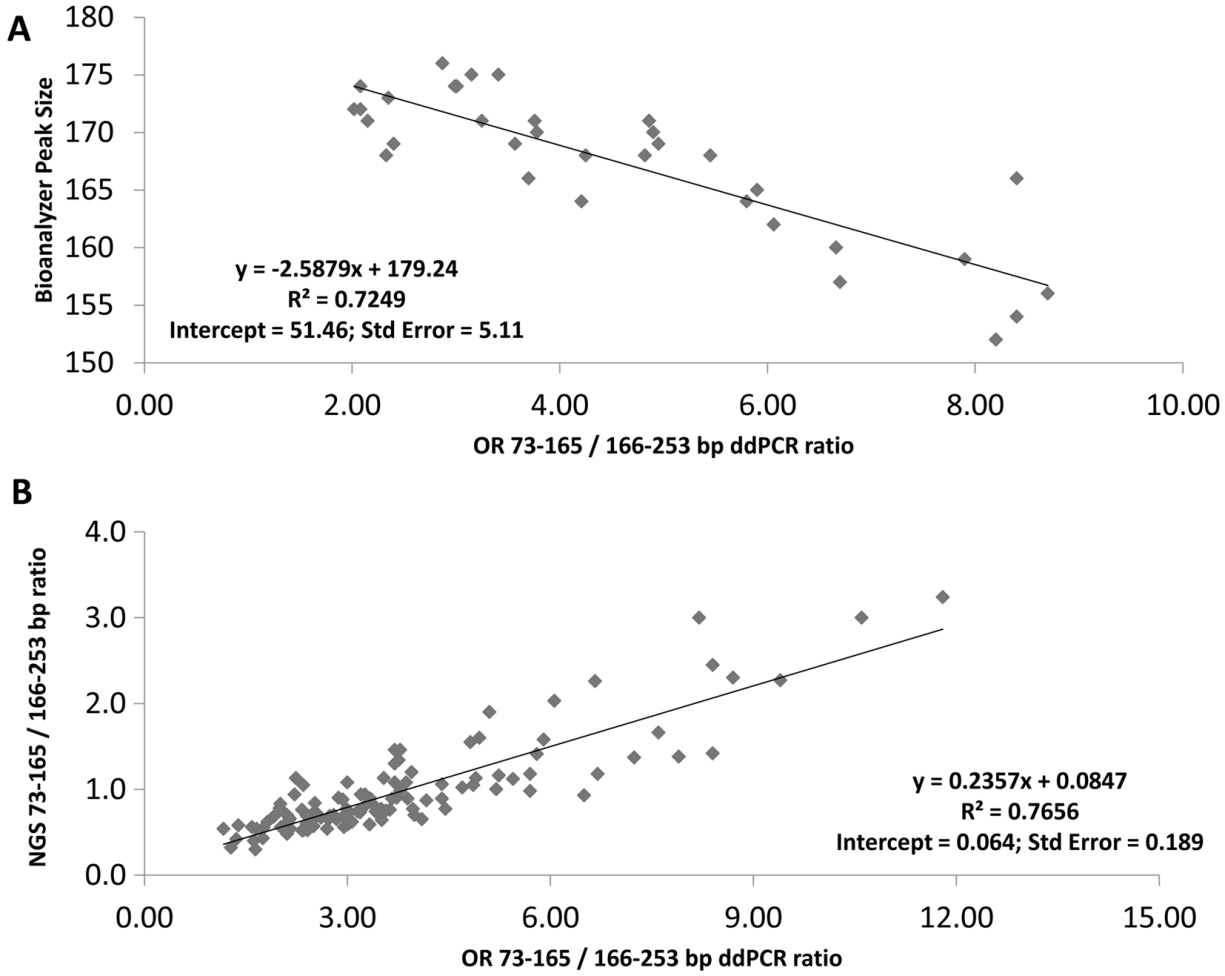
Correlation between cfDNA size distribution estimates determined by ddPCR and other approaches. (**A**) Estimated cfDNA size distributions inferred from ddPCR is compared to the electrophoretic mobility of cfDNA fragments. (**B**) The same values are compared to cfDNA size distributions determined from the analysis of the insert sizes of cfDNA-derived libraries. A strong outlier showing the highest ratio of short to medium-sized cfDNA fragments in both our ddPCR estimate (18.0) and the inspection of high throughput sequencing reads (10.4) was excluded from Panel B for visualization purposes.

Our assay provided visual clues regarding cfDNA fragment size distributions that could be compared with those generated by electrophoretic and high throughput sequencing methods (Fig. 2). The average ratio between short (73–165 bp) and medium-sized (166–253 bp) cfDNA fragments across 117 tested samples was 3.87 ± 2.41 S.D.; minimum = 1.17; maximum = 18.0). The fractional abundance of fragments longer than 253 bp ranged from 1.12 to 40.90% (average = 11.69% ± 8.61% S.D.). These analyses excluded samples obviously contaminated with gDNA (see below). Repeated assays conducted on pooled cfDNA from healthy donors reported short to medium-sized ratios ranging between 1.0 and 1.20 and fractions of fragments > 253 bp around 25–30%. Highly fragmented cfDNA samples exhibited relatively lower droplet counts for both medium-sized and long cfDNA fragments (see Fig. 2, Panel C). The progressive dilution of one sample exhibiting short cfDNA fragments using cfDNA from another “normal” sample carrying longer fragments showed strong linearity (R^2^ = 0.884, P < 0.001, N = 7; Figure S1). Notably, we also observed a significant correlation between our size distribution estimates (short to medium-sized ratio) and the observed size (in bp) of the highest peak during the electrophoretic separation of cfDNA fragments (R^2^ = 0.725, P < 0.001, N = 34; Fig. 3A). Similarly, we observed a significant correlation between our estimates of fragment size distribution and those relying on the analysis of high throughput sequencing data (R^2^ = 0.766, P < 0.001, N = 115; Fig. 3B).

We conducted our assay using different inputs (0.30 to 12.45 ng) from the same cfDNA sample (Table 1). Although assay precision was substantially influenced by cfDNA input, we observed that the ratio of short to medium-sized OR fragments did not differ in more than 10% from the averaged value when investigating cfDNA inputs below 5 ng (average = 3.80; maximum = 4.03, minimum = 3.66). We nonetheless observed that using cfDNA inputs above 5 ng translated into comparably lower ratios of short to medium-sized OR fragments (Table 1, see discussion). We generated two replicates from 51 cfDNA extracts to assess the reproducibility of our assay. Notably, we observed that each replicate did not differ in more than 5% from the average value calculated from both replicates in 33 samples (64.7%), 6 to 10% in 15 samples (29.4%) and between 11–15% in only 3 samples (5.9%). All samples investigated were in agreement with cfDNA inputs above 1 ng per replicate, according to our inferences based on the number of positive droplets for the *STAT6* locus.

**Table 1:** Ratio between short (73–165 bp) and medium-sized (166–253 bp) OR fragments when using variable inputs from the same cfDNA sample in ddPCR reactions. Inputs below 5 ng generate reasonably similar ratios after analysing two assay replicates per sample. The short to medium-sized OR ratio nonetheless starts deflating for cfDNA inputs above 5 ng (see discussion). The ratio calculated from the 12.45 ng input is the only one that is significantly different (p < 0.001) from the other calculated ratios.

cfDNA INPUT (ng)	OR short/medium ratio	Poisson max ratio	Poisson min ratio
0.30	3.70	4.20	3.10
0.42	3.66	4.12	3.19
0.72	4.00	4.43	3.57
1.66	3.67	3.92	3.42
3.80	3.72	4.08	3.38
4.70	4.03	4.21	3.85
6.25	3.22	3.34	3.09
12.45	2.47	2.54	3.39

We also independently explored the size distribution of cfDNA fragments in 600 libraries built from human plasma in an effort to corroborate the observed relationship between altered cfDNA size distributions and ctDNA levels. Our results indeed confirmed a trend pointing towards higher degree of cfDNA fragmentation in samples with high ctDNA content (Figure S2). By considering the average size distribution ratios for each range of the following estimators of ctDNA abundance (0% ctDNA, 0.1–10% ctDNA, 10–25% ctDNA, > 25% ctDNA), our data supports the fact that the extent of cfDNA fragmentation can be used to some extent to predict ctDNA levels (Table S1). As we found a significant correlation between our ddPCR estimates and those relying on high-throughput sequencing estimates (Fig. 3), we believe our assay has the potential to stratify cfDNA samples based on presumed ctDNA levels in a faster and more cost-effective way. However, we occasionally observed highly fragmented cfDNA extracts showing no trace of ctDNA as well as plasma samples carrying high levels of ctDNA but exhibiting normal size distribution profiles in our cohort. To explore this further, we tracked the evolution of fragment size ratios across different plasma samples obtained from the same patients but drawn at different time points during the course of their therapeutic interventions. While fragment size ratios mimicked quite well the evolution of the allele frequency of somatic mutations in many cases (Figure S3) we also observed scenarios were these acted as poor predictors of patient response or disease progression (Figure S4).

### Detecting cfDNA extracts contaminated with gDNA and/or PCR inhibitors

**Figure 4:**
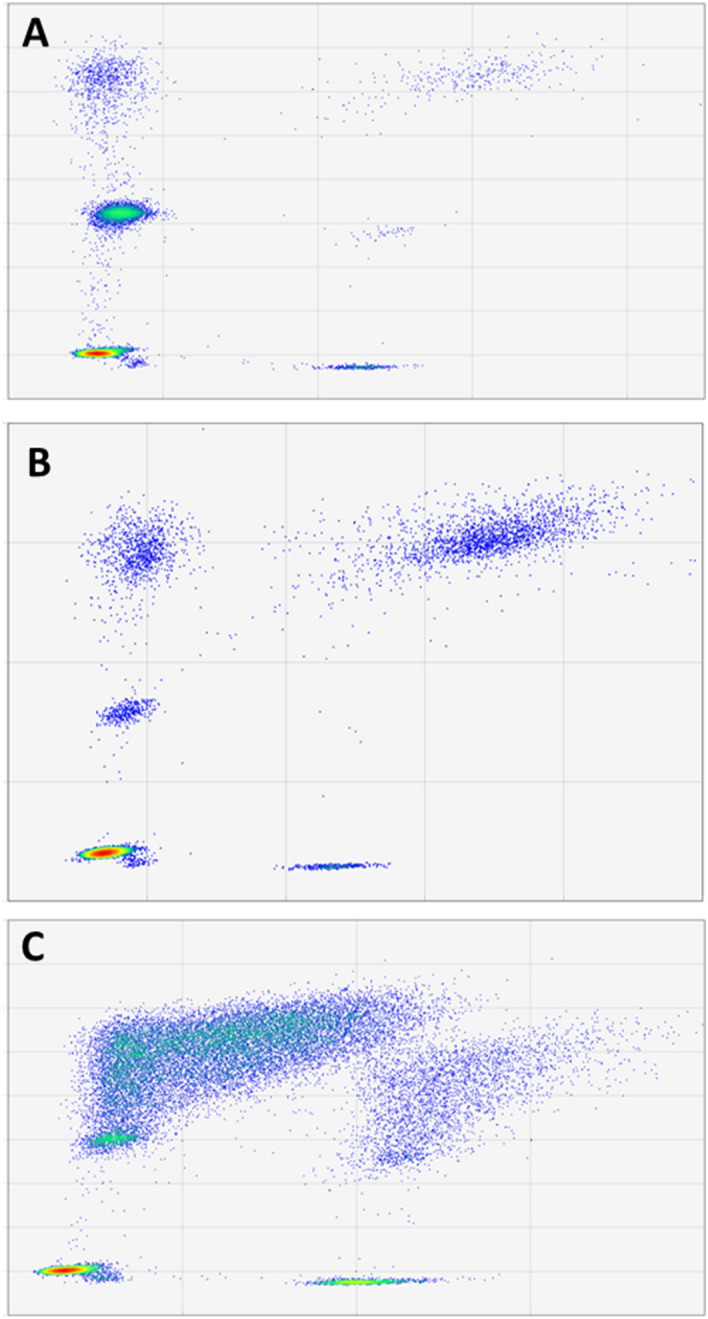
Comparison of high quality cfDNA samples to samples with high molecular weight DNA contamination. Two-dimensional fluorescence plots associated with a normal cfDNA sample (**A**), a cfDNA extraction with unusually high levels of long cfDNA fragments (**B**) and a highly concentrated cfDNA sample exhibiting significant levels of high molecular weight DNA contamination (**C**). Unusual fragmentation patterns, where cfDNA is more commonly wrapped around 2 or more nucleosomes or its mostly originating from necrotic rather than from apoptotic cells, may exhibit aberrantly large cfDNA size distribution profiles even after performing size selection.

The presence of gDNA contamination in cfDNA extracts produced unusually high fractions of OR fragments > 253 bp (> 50% of total fragments) and short to medium-sized OR ratios below 1.0 (see Fig. 4). Such pattern was commonly observed in blood samples preserved in Streck tubes but not processed within the first 14 days after collection. Double size-selection proved useful to decrease the proportion of OR cfDNA fragments longer than 253 bp in samples exhibiting significant levels of gDNA contamination. It must be noted that size-selection steps inevitable cause a significant decrease in total cfDNA yields and therefore should be reserved for situations when the presence of high levels of gDNA contamination may have a significant negative impact in downstream analyses (see Figure S5). We also observed comparably higher short to medium-sized OR ratios when this strategy was applied to a random set of samples not showing evidence of gDNA contamination (Table S2). The presence of PCR inhibitors, on the other hand, precluded an adequate formation and separation of the four fluorescence clusters shown in Figs. 1 and 2 (panels B and C). In essence, we observed poor separation between the two clusters formed across the FAM channel and very low droplet counts for fragments longer than 253 bp, probably because inhibitors had a great effect on longer amplicons. We nevertheless could ameliorate the detrimental effect of PCR inhibitors by applying a single round of sample purification using 2.0 × volumes of magnetic beads (Figure S6).

## Discussion

In the assay described herein, we have leveraged the potential offered by ddPCR to implement a generic assay for estimating the absolute concentration, quality and size distribution of cfDNA samples. All solid tumours and healthy tissues are expected to shed variable amounts of cfDNA into the bloodstream that can be characterized and quantified by our ddPCR assay. However, aberrant cfDNA size distributions associated with high levels of ctDNA are less likely to be detected for those tumours affecting the central nervous system, owing to the fact that the blood–brain barrier prevents ctDNA to reach peripheral circulation. We show that our assay yields robust results with just 1–3 ng of cfDNA but it must be noted that the determination of cfDNA yields can be confounded by copy number alterations affecting the control diploid locus of choice and by the extent of gDNA contamination. Such contamination may greatly dilute clinically relevant cfDNA and undermine downstream analysis, particularly those relying on the relative frequencies of somatic mutations or those that need to start from fixed cfDNA inputs^3,15,27,28^. DNA fragments longer than 1 kb, however, cannot be successfully incorporated into libraries and gDNA contamination is not critical for those cases reporting mutant molecules detected per volume of sample^3^. An adequate handling of biofluid specimens can in any case prevent this source of contamination but, where this is not possible, it can be detected and addressed by selection using, for example, magnetic beads^3,13,15,27^. If high fractions of long cfDNA fragments persists even after size selection, this may support the occurrence of aberrant cfDNA profiles probably linked to extensive necrosis^7^.

Besides the fact that ddPCR is considered more resilience to inhibitors than qPCR, our assay can detect their presence without the need for spiked-in reference samples^14,15^. Enzymatic inhibitors can interfere with library construction and negatively impact the yield, diversity and introduce biases in library composition^15^. Inhibitors may have even a more detrimental effect during the application of PCR-based methods^24^. Some specialized ddPCR approaches (e.g. “wild-type” negative, highly multiplexed hotspot or copy number assays^29–32^, for example, rely on optimal separation of multiple fluorescence clusters and poor amplification efficiency of some of the targets can affect assay performance by increasing the amount of ddPCR noise (“rain”). As these assays are often conducted on clinically precious and limiting DNA samples, cfDNA QC is imperative to avoid generating low quality or unusable data. The detrimental effect of inhibitors can nevertheless be reverted by conducting simple purifications using magnetic beads. This re-extraction method has been previously shown to be more effective than in silica membrane-based methods when removing sample impurities^15^. Additional sample purification steps are nevertheless expected to alter fragment size distributions and cause signification losses of total cfDNA yields, and hence, must be reserved for situations that warrant this extreme measure. Unquestionably, the whole process stemming from sample collection to cfDNA elution must be conducted as homogeneously as possible to generate comparable fragment size distribution estimates. In this regard, we find encouraging that our data strongly corroborates the association between ctDNA levels and cfDNA fragment size distributions in spite of the analysis of blood samples processed and manipulated using different methods. Therefore, we expect future studies homogenizing sample processing and extraction steps to obtain even better results than the ones here described.

Following the investigation of cfDNA fragmentation profiles, we believe our assay enables better data normalization by mostly focusing on the size distribution within the major cfDNA peak^3,8^. Sample partition minimizes possible competition between PCR amplicons of different sizes, then enabling more accurate estimates and the investigation of three range sizes in one single experiment. Since ctDNA mostly differs from the rest of cfDNA by the subtle presence or absence of the linker DNA^18,20^, and this could be captured by our 73–165 bp / 166–253 bp ratio, our assay avoids the interfering of longer DNA fragment of multiple sources or linked to different causes during size distribution estimations. Samples exhibiting a strong bias towards very short cfDNA fragments, for example, might be considered better suited to single strand library preparation methods^33,34^ and/or encourage the design of ultra-short amplicons^3,15^. Contrarily to ddPCR-based assays relying on EvaGreen chemistry^35^, our assay can be conducted in one single well using low cfDNA inputs. In fact, inputs larger than 5 ng may underestimate short cfDNA fragments and should be avoided. Considering the average cross-amplification of 10 OR loci in our assay, this equates to an expectation of 3,030 OR copies/ng of cfDNA. Hence, using 5 ng of cfDNA may exceed the number of OR copies with respect to the total number of droplets that can be generated by the QX200 platform (~ 15,000). Those droplets carrying both small and medium-sized OR fragments will emit fluorescence at the same intensity as droplets containing only medium-sized cfDNA fragments. While qPCR assays, particularly those relying on the amplification of repetitive regions of the genome^22,36^, are not constrained by cfDNA inputs, these assays may be more sensitive to gDNA contamination. This source of contamination may destabilize size distribution calculations for those ddPCR assays that exclusively interrogate two DNA fragment ranges as well^37^. Moreover, shorter amplicons are more likely to outcompete longer amplicons if they share the same partition because the more favorable replication of the former in such designs.

One potential issue of our assay can be caused by deletions of chr7q35. Such deletions would mitigate (heterozygous deletions) or totally obscure (homozygous deletions) cfDNA fragmentation patterns originating in tumours because our primers will mostly replicate OR genes in cfDNA shed by non-cancerous cells. That said, the inspection of copy number profiles affecting these OR genes in the cosmic database (e.g. https://cancer.sanger.ac.uk/cosmic/gene/analysis?ln=OR2A7;https://cancer.sanger.ac.uk/cosmic/gene/analysis?ln=OR2A1; https://cancer.sanger.ac.uk/cosmic/gene/analysis?ln=OR2A42; https://cancer.sanger.ac.uk/cosmic/gene/analysis?ln=OR2A4; https://cancer.sanger.ac.uk/cosmic/gene/analysis?ln=OR2A25) supports the fact that copy number gains are significantly more common than losses. Rare germline deletions of chr7q35^38^, on the other hand, could affect assay precision. Our ddPCR-based estimates correlate well with electrophoretic and sequencing data estimates, suggesting OR copy number losses should only be rarely encountered. It is also notable that the short-to-medium size ratio estimated from our ddPCR data deviates significantly in magnitude relative to the size ratio observed from the analysis of sequencing data (Fig. 3). We speculate that this is caused by small cfDNA fragments having difficulty to be incorporated into cfDNA libraries constructed with standard dsDNA ligation-based library prep methods, as suggested by recent studies exploring the potential of single stranded library preparation^33,34^.

Finally, we have corroborated^10,18,20^ a trend showing higher ctDNA content in samples showing shorter cfDNA size distributions (Figure S2). This information can be useful to stratify liquid biopsies based on anticipated ctDNA levels, without any a priori information regarding the somatic mutations occurring in any given patient, and then envisage sequencing strategies accordingly. For example, samples with low ctDNA content would need deeper sequencing than those presumably high in ctDNA. Samples showing a bias towards small fragment sizes could also be considered suitable for whole exome or genome sequencing (Figure S5). That being said, we have also observed samples with very high ctDNA content but having size distribution profiles that would be more consistent with very low (or absent) ctDNA. On the other hand, we have observed samples with undetectable ctDNA that exhibited highly fragmented cfDNA profiles, particular in some patients that recently underwent surgical interventions or were subjected to extensive radiation sessions. Several studies have certainly started exploring the diagnostic and prognostic value of cfDNA fragmentation profiles across different cancer types^22,26^. Our anecdotal analysis on a restricted number of patients supports the notion that cfDNA size distributions can be misleading in certain scenarios and future studies must investigate in more depth this potentially confounding factor. Taken together, our data show that assays inferring ctDNA levels from cfDNA size distributions are a convenient approach to identify candidate samples with high ctDNA but these measurements must be always corroborated by complementary methods.

## Methods

### Assay design

We designed one reverse primer targeting a conserved region of seven human olfactory receptor genes^39^. Six of these olfactory receptor genes are located in chromosome 7q35 (*OR2A7, OR2A1, OR2A42, OR2A20P*, *OR2A9P and OR2A25*) and one of them (*OR2A4*) maps to chromosome 6q23.2. We then designed three forward primers with the aim to generate three distinct amplicon populations. The concentration of these forward primers was adjusted such that longer amplicons could be preferentially amplified over short amplicons (Table S3). We also strategically designed three hydrolysis probes intended to report the presence of cfDNA fragments of different sizes. We leveraged the power of sample partition offered by ddPCR to facilitate the isolation of individual cfDNA fragments within droplets (see Fig. 1 for details). The fourth hydrolysis probe should target any diploid locus in the genome, preferentially one gene not commonly affected by copy number variations. In this paper, we used a probe targeting *STAT6* because of its availability in our lab. OR primers and probes were manufactured by Integrated DNA Technologies (Neward, NJ, USA), the second as 100 nM PrimeTime double-quenched probes. Hydrolysis probes were resuspended at 100 µM using low EDTA TE buffer and used at different final concentrations in the ddPCR reaction, with the goal to generate a higher fluorescence signal for longer cfDNA fragments. A 20 × pre-mix of primers and probes (1:1 primers/probe ratio) for the *STAT6* locus was ordered from Bio-Rad (Hercules, CA, USA). This probe can generate secondary low-fluorescence clusters if somatic mutations in *STAT6* (codon 419) occur^29^. Because of design constrains, only the hydrolysis probe aimed at reporting the existence of fragments > 253 bp overlaps with known single nucleotide polymorphisms in two of the OR genes (rs199675686, rs131701146, rs6173133697). The frequency of the minor allele for these polymorphisms is nevertheless below 15%. In the absence of a probe competing for hybridization, it has been demonstrated that hydrolysis probes can be degraded in spite of 1–2 mismatches with respect to the DNA template, but will generate a lower fluorescence signal^29^.

### Droplet digital reactions and statistical analyses

We investigated the size distribution of cfDNA samples by calculating the ratio between short (73–165 bp) and medium-sized (165-253 bp) OR fragments. The fractional abundance of fragments longer than 253 bp was also calculated but not accounted for during our cfDNA size distribution calculations because of the confounding effect of gDNA contamination, which may vary between different cfDNA samples. The setup of ddPCR reactions is detailed in the legend of Table S3. ddPCR reactions were set up in a final volume of 22 µl containing 11 µl of the ddPCR Supermix for probes (no dUTP) (Bio-Rad). We loaded a variable amount of cfDNA (between 1–3 ng, when possible) and volume of ultra-pure water to fill up the rest of the reaction after having added all primers, probes and ddPCR Supermix (Table S3). Droplets were generated in an automated droplet generator (ddPCR QX-200 system, Bio-Rad) and thermocycled according to the following protocol: 10 min at 95 °C followed by 80 cycles of 30 s at 94 °C and 90 s at 58 °C, with a final step of 10 min at 98 °C. A total number of 80 cycles provides the best separation of clusters and minimize digital PCR “rain” but the assay is expected to also perform well with lower number of cycles (e.g. 45–60). Droplets were kept at 4 °C until their analysis in an automated droplet reader (Bio-Rad). Raw data was inspected and analyzed using the QuantaSoft Analysis Pro Edition software ver 1.0.596 (Bio-Rad). Regression analyses to evaluate the correlation between our ddPCR-based estimates of fragment size distribution and those calculated from the analysis of sequencing data and the electrophoretic mobility of cfDNA fragments were performed in Microsoft Excel 10. Other statistical analyses were performed in R.

### Human subjects, plasma and cfDNA processing

We applied our ddPCR assay to a collection of 117 plasma samples obtained from patients diagnosed with Non-Hodgkin lymphoma and prostate cancer (Table S4). Blood samples were handled at different laboratories following slightly different protocols, but in essence, plasma was separated from the rest of blood by centrifugation and then kept at – 80 °C until further processing. Blood was either preserved in Cell-free DNA BCT (Streck) tubes and centrifuged within 2 weeks (when possible). Alternatively, the plasma fraction was isolated within 4 h after the blood draw when using EDTA collection tubes. All patients provided written, informed consent. This project was approved by the research ethics boards at the Jewish General Hospital, British Columbia Cancer Agency, British Columbia Children’s Hospital, Simon Fraser University and is in accordance with the declaration of Helsinki. We also used pooled human plasma from healthy donors (apheresis-derived, Cat. No. IPLAK2E10ML, Innovative research, Novi, MI, USA) as a control to estimate cfDNA size distributions in the absence of disease. Total cfDNA was extracted according to different protocols and commercially available kits, including the MagMAX Cell-Free DNA Isolation Kit (ThermoFisher Scientific, Waltham, MA, USA) or the QIAamp Circulating Nucleic Acid Kit (Qiagen, Hilden, Germany). The concentration of the cfDNA extracts was estimated using the Qubit dsDNA HS Assay Kit (ThermoFisher Scientific). The fragment size distribution for a subset of cfDNA extracts (N = 34) was investigated using High Sensitivity DNA chips ran in an Agilent 2,100 BioAnalyzer (Agilent, Santa Clara, CA, USA). Those samples suspected to be contaminated with PCR inhibitors were subjected to a round of sample clean up using 2.0 × volumes of Agencourt AMPure XP magnetic beads. (Beckman-Coulter, Brea, CA, USA) and two 80% ethanol washes. We also performed a double size selection (0.5 × /2.0 ×) using AMPure XP magnetic beads in a subset of samples suspected to be contaminated with gDNA.

Our validation cohort of 117 samples belonged to larger group of 600 cfDNA samples in which we also investigated cfDNA size distribution by Illumina sequencing (Table S4). In essence, cfDNA libraries were constructed and enriched using a custom panel of biotinylated baits as described previously^40^. Raw reads were mapped against a reference encompassing a specific array of disease-specific genes using Geneious ver 9.1.5. PCR and optical duplicates were removed using MarkDuplicates (https://broadinstitute.github.io/picard/). For each library, we calculated the ratio of small cfDNA fragments (73–165 bp) to mid-range fragments (166—253 bp). The ctDNA content for each of these libraries was separately estimated by averaging the variant allele frequencies (VAF) of the three most abundant somatic mutations detected in the cfDNA library. A sample was considered ctDNA negative if we could not find support for at least two variants previously identified in a matched tumour sample or in a different cfDNA sample obtained from the same patient.

## Supplementary information

Supplementary information.
