# Supplementary material for: Evaluating the quantity, quality and size distribution of cell-free DNA by multiplex droplet digital PCR
Source: Sci Rep. 2020 Jul 28;10:12564. doi: 10.1038/s41598-020-69432-x (PMC7387491; doi:10.1038/s41598-020-69432-x)
Supplement: Supplementary file 1 — Supplementary information. [file 41598_2020_69432_MOESM1_ESM.docx]

**SUPPLEMENTAL INFORMATION**

**Evaluating the quantity, quality and size distribution of cell-free DNA by multiplex droplet digital PCR**

**Miguel Alcaide^1^, Matthew Cheung^1^, Jack Hillman^1^, S. Rod Rassekh^2^, Rebecca J. Deyell^2^, Gerald Batist^3^, Aly Karsan^4^, Alexander W. Wyatt^5^, Nathalie Johnson^6^, David Scott^7^ and Ryan D. Morin^1*^**

| **Fragment ratio (73-165 vs 166-253 bp)** | **% of samples that are ctDNA +** | **% of samples that contain at least 50% ctDNA** | **N** |
| --- | --- | --- | --- |
| >0.59 (0% ctDNA) | 85.21% | 32.33% | 399 |
| >0.70 (0.1-10% VAFs) | 89.96% | 39.28% | 308 |
| >0.86 (10-25% VAFs) | 94.68% | 50.26% | 187 |
| >1.12 (>25% VAFs) | 94.18% | 65.88% | 85 |

**Table S1. The analysis of the size distribution of sequencing reads can be useful to predict ctDNA levels in liquid biopsy specimens.** Here, the vast majority of samples (85.21%) showing fragment ratios above the average 73-165/166-253 bp ratio calculated from samples that were deemed negative for ctDNA, were ctDNA positive. About two thirds (65.88%) of the samples with fragment ratios above the averaged ratio calculated from libraries estimated to contain more than 50% ctDNA (based on the relative frequency or VAFs of known somatic mutations in plasma) exhibited ctDNA levels above 50%. The number of individual libraries (N) used during the calculations of the average 73-165/166-253 bp ratio for each category is shown on the right column. Please note these ratios were calculated from sequencing and not ddPCR data (see also Figure S2).

|  | **OR Short/Medium Ratio** | **p-value** |
| --- | --- | --- |
| Sample 1 | 3.72 [4.08,3.38] / 4.41 [4.69, 3.80] | 0.024 |
| Sample 2 | 1.66 [1.81,1.50] / 2.52 [3.20,1.90] | 0.002 |
| Sample 3 | 8.40 [9.40, 7.50] / 9.90 [11.10, 10.80] | <0.001 |
| Sample 4 | 4.85 [5.21, 4.48] / 5.43 [5.83, 5.02] | 0.012 |

**Table S2. Ratios between short and medium-sized OR fragments in cfDNA extracts before (eft) and after (right) double size-selection with magnetic AMPure XP beads (0.5x / 2.0x volumes, respectively).** We conducted two replicates per sample. Poisson confidence intervals and p-values are also provided.

| **Primer/Probe Name** | **Primer/Probe Sequence** | **Final concentration** |
| --- | --- | --- |
| OR7x-Rv | 5'-AGAGTTCCTCCTACTGGG-3' | 1.14 µM |
| OR7x-Fw1 | 5'-GAACAGGGAGAAGAGCC-3' | 0.57 µM |
| OR7x-Fw2 | 5'-TGTGAGAGGAAGAAGTACATG-3' | 0.76 µM |
| OR7x-Fw3 | 5'-AAAGGAGATGGGCTTGG-3' | 1.70 µM |
| OR7x-Short | 5'-/56FAM/AGCATCTGA/ZEN/ATCCTTGGGCC/3IABkFQ/-3' | 0.14 µM |
| OR7x-Medium | 5'-/56FAM/TCCAGTGA/ZEN/GATGAGCCCCA/3IABkFQ/-3' | 0.54 µM |
| OR7X-Long | 5'-/HEX/TGCAGGAGG/ZEN/TTCRCCAGC/3IABkFQ/-3' | 0.68 µM |
| STAT6-Fw | 5'-AGGCATTGTCCCACAG-3' | 0.91X |
| STAT6-Rv | 5'-CCTGGTGGTCATCGTC-3' | 0.91X |
| STAT6-D419 | 5'-/HEX/TGGCATTGTTGTCTTGGTTG/3IABkFQ/-3' | 0.91X |

**Table S3. DNA sequences of the primers and probes involved in our multiplexed ddPCR assay to estimate the quality, quantity and fragment size distribution of cfDNA extracts. The final concentration of each oligonucleotide in the ddPCR reaction is also indicated.** An adequate cluster resolution is critical for a good performance of our assay. Implementation of our method may require fine-tuning the annealing temperature as well as the final concentration of primers and probes if significant differences are observed with respect to the 2D ddPCR plots shown in this article.

| **Disease** | **Fragment size distribution in NGS libraries** | **Fragment size distribution estimated from ddPCR assay** |
| --- | --- | --- |
| Non-Hodgkin Lymphoma | 402 | 94 |
| Hodgkin Lymphoma | 15 | 0 |
| Lung Cancer | 24 | 0 |
| Pancreatic Cancer | 7 | 0 |
| Colorectal Cancer | 52 | 0 |
| Prostate Cancer | 23 | 23 |
| Other Cancers | 77 | 0 |

**Table S4. Summary of the disease diagnosis for all plasma samples investigated in this study either through the analysis of cfDNA-derived libraries subjected to high throughput sequencing or via the application of our novel ddPCR assay.**


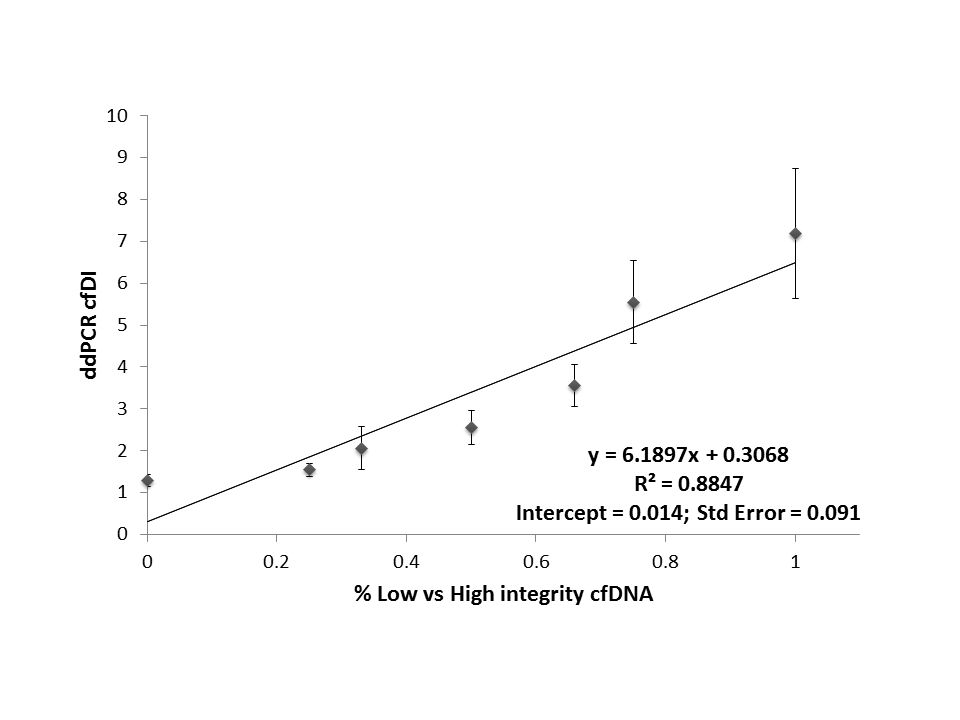


**FIGURE S1. Progressive dilution of a cfDNA sample showing a bias towards very small cfDNA fragments with cfDNA from another sample showing a “normal” cfDNA fragment size distribution.** We prepared stocks of the two samples at the same concentration of 1 ng/µL and prepared 3:1, 2:1, 1:1, 1:2 and 1:3 dilutions (X-axis). Our 73-165bp/166-253 bp ddPCR ratio is plotted on the Y axis. Poisson confidence intervals for each serial dilution point are shown.


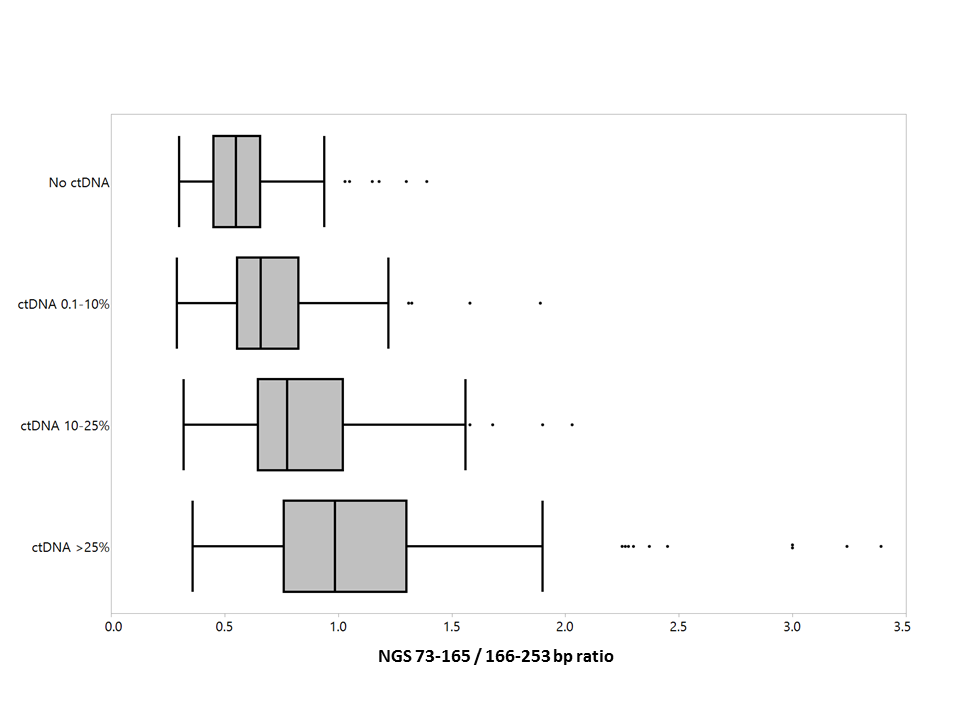


**FIGURE S2. Insert size distributions of cfDNA-derived libraries.** Using available data from 600 cancer patients, we have corroborated a trend towards smaller cfDNA size distributions in the plasma of cancer patients with high levels of circulating tumour DNA, as determined via the calculation of the variant allele frequency of somatic mutations (Methods). A single outlier with extreme cfDNA fragmentation but no ctDNA is excluded from this figure (see also Figure 3).


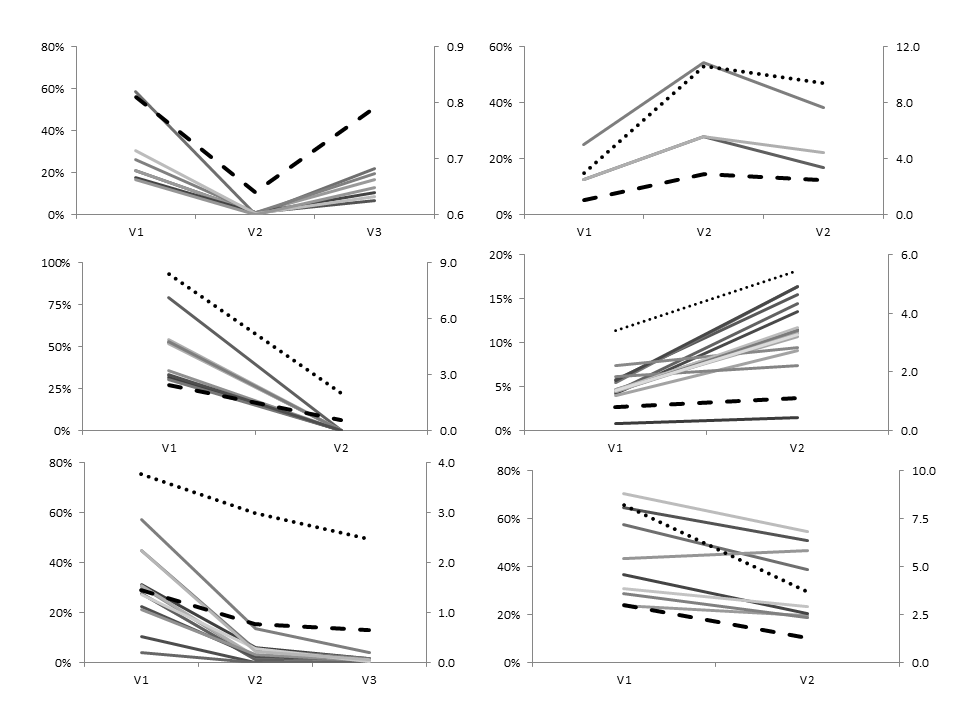


**FIGURE S3. Aberrant cfDNA size distributions often track with the allele frequencies of known somatic mutations in ctDNA.** This panel summarizes the analysis of cfDNA size distributions in six cancer patients diagnosed with colorectal, prostate cancer or diffuse large B-cell lymphoma across several plasma samples obtained at different stages during therapeutic interventions (V1 to V3). Solid lines represent the allele frequency (% in left Y axis) of multiple somatic mutations. Dashed lines indicate cfDNA size distribution ratios (73-165bp vs 166-253bp; right Y axis) calculated from the analysis of cfDNA-derived libraries. Dotted lines represent the estimated cfDNA size distribution values calculated from our multiplex ddPCR assay in the same set of samples. A decrease in the allele frequencies of somatic mutations and an increase in the integrity of cfDNA samples are in agreement with some extent of clinical response. An increase in the allele frequencies of somatic mutations, together with the observation of still highly or even more fragmented cfDNA profiles, support disease progression or relapse.


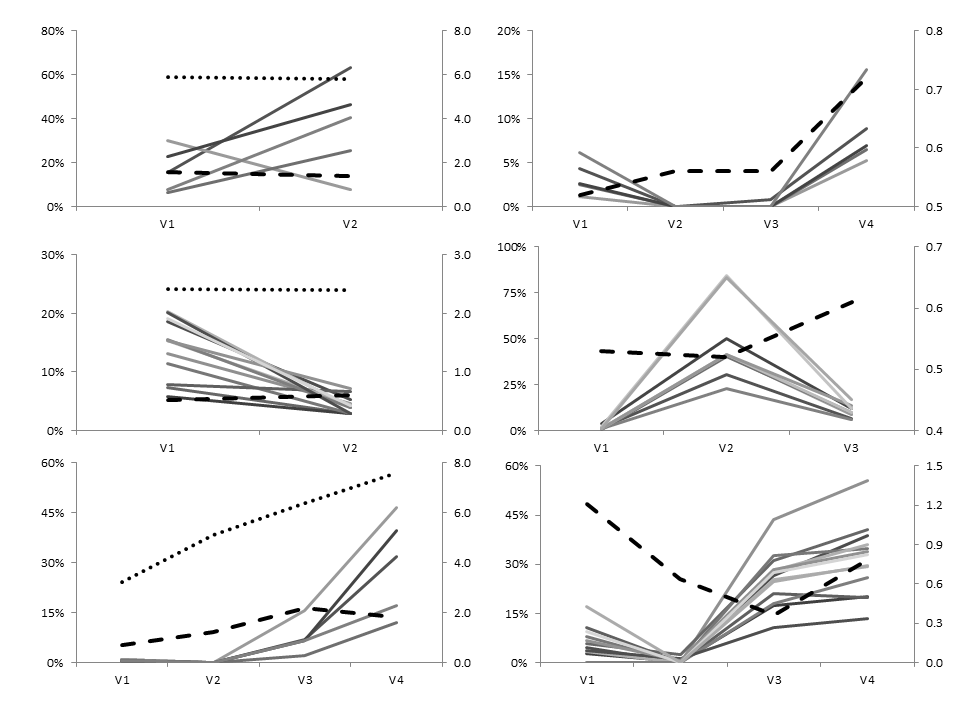


**FIGURE S4. Exceptions exhibiting discordant cfDNA fragment distribution profiles and variant allele frequencies in ctDNA.** This panel summarizes the analysis of cfDNA size distributions in five patients diagnosed with diffuse large B-cell lymphoma and one patient diagnosed with colorectal cancer across several plasma samples obtained at different stages during therapeutic interventions (V1 to V3). The first panel on the left shows an example where cfDNA size distributions show limited fluctuation between pre and the post-treatment samples in spite of a significant increase in the allele frequency of multiple somatic mutations. In the middle-left panel, a patient exhibits a significant decrease in the allele frequencies of multiple somatic mutations that is not captured by fragment size distribution values. In the bottom-left panel, a surgical intervention (V2) in a colorectal cancer patient likely caused the release of highly fragment cfDNA from non-cancerous cells that confounded the signal associated with the release of ctDNA. In the top-right panel, cfDNA size distribution profiles fail to capture initial clinical response but supports relapse later on. Finally, the last two examples illustrate cases where cfDNA size distribution profiles are poor predictors of the course of the disease and do not align with the evolution of the allele frequencies of somatic mutations.


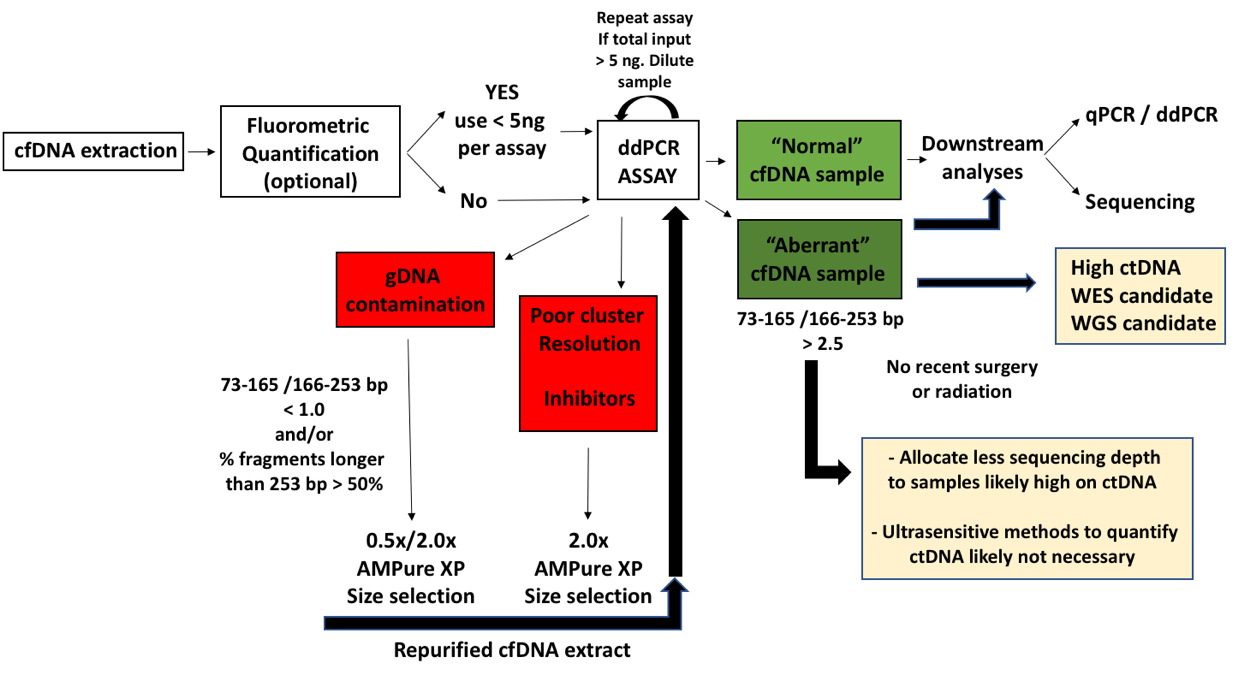


**FIGURE S5**. Analytical workflow for the QC of cfDNA samples using the ddPCR assay described herein. Our assay may enable researchers and clinicians to select samples presumably high on ctDNA based on the observation of aberrant size distribution profiles in cfDNA. Such samples could be ideal for whole exome and/or genome sequencing and may require lower number of reads during targeted sequencing experiments. Our assay also allows the detection of samples contaminated with high molecular weight DNA and PCR inhibitors, which detrimental effect on downstream analyses can be reverted or minimized by carrying out additional sample purification steps.


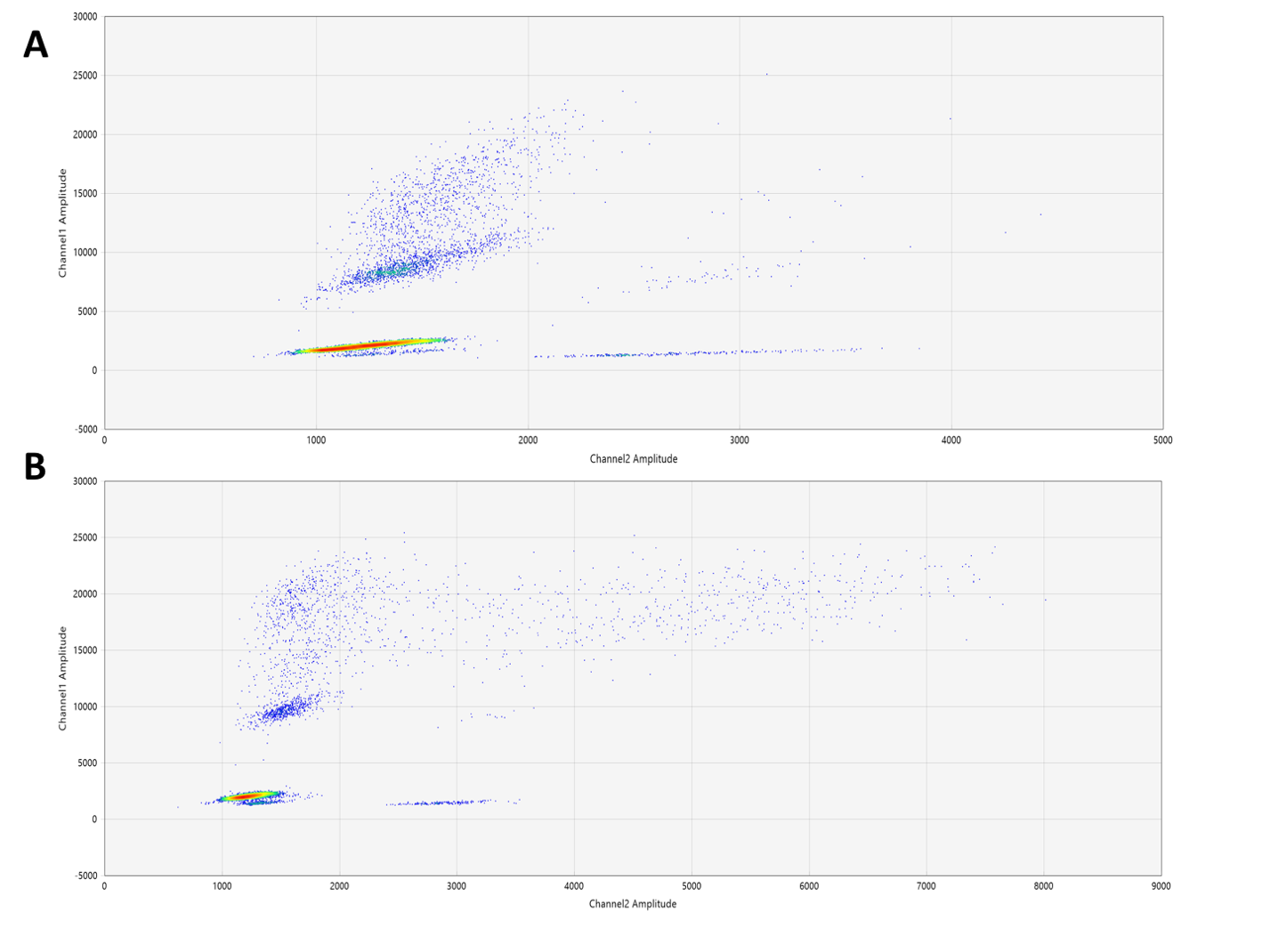


**FIGURE S6. Post-extraction sample purification steps using magnetic beads may substantially improve assay performance.** The presence of PCR inhibitors in this particular cfDNA sample not only generated poor cluster separation in panel A but also precluded the replication and reporting of fragments longer than 253 bp. Enhanced cluster differentiation, together with the successful quantification of long cfDNA fragments, is achieved when the sample was subjected to an additional round of sample purification using magnetic beads (Panel B).
